# Effects of turbidity, temperature and predation cue on the stress response of juvenile delta smelt

**DOI:** 10.1093/conphys/coad036

**Published:** 2023-06-26

**Authors:** Christina Pasparakis, Toni Lohroff, Felix Biefel, Dennis E Cocherell, Evan W Carson, Tien-Chieh Hung, Richard E Connon, Nann A Fangue, Anne E Todgham

**Affiliations:** Department of Environmental Toxicology, University of California Davis, 1 Shields Ave., Davis, CA, USA; Bodega Marine Laboratory, University of California Davis, 2099 Westshore Rd., Bodega Bay, CA, USA; Department of Wildlife, Fish and Conservation Biology, University of California Davis, 1 Shields Ave., Davis, CA, USA; Department of Animal Science, University of California Davis, 1 Shields Ave., Davis, CA, USA; School of Veterinary Medicine, Department of Anatomy, Physiology and Cell Biology, University of California Davis, 1 Shields Ave., Davis, CA, USA; Department of Wildlife, Fish and Conservation Biology, University of California Davis, 1 Shields Ave., Davis, CA, USA; San Francisco Bay-Delta Fish and Wildlife Office, U.S. Fish and Wildlife Service, 650 Capitol Mall, Sacramento, CA, USA; Fish Conservation and Culture Laboratory, Department of Biological and Agricultural Engineering, University of California Davis, 1 Shields Ave., Davis, CA, USA; School of Veterinary Medicine, Department of Anatomy, Physiology and Cell Biology, University of California Davis, 1 Shields Ave., Davis, CA, USA; Department of Wildlife, Fish and Conservation Biology, University of California Davis, 1 Shields Ave., Davis, CA, USA; Department of Animal Science, University of California Davis, 1 Shields Ave., Davis, CA, USA

**Keywords:** supplementation, San Francisco Estuary, delta smelt, conservation, climate change

## Abstract

The San Francisco Estuary (SFE) is one of the most degraded ecosystems in the United States, and organisms that inhabit it are exposed to a suite of environmental stressors. The delta smelt (*Hypomesus transpacificus*), a small semi-anadromous fish endemic to the SFE and considered an indicator species, is close to extinction in the wild. The goal of this study was to investigate how environmental alterations to the SFE, such as reductions in turbidities, higher temperatures and increased prevalence of invasive predators affect the physiology and stress response of juvenile delta smelt. Juvenile delta smelt were exposed to two temperatures (17 and 21°C) and two turbidities (1–2 and 10–11 NTU) for 2 weeks. After the first week of exposure, delta smelt were exposed to a largemouth bass (*Micropterus salmoides*) predator cue at the same time every day for 7 days. Fish were measured and sampled on the first (acute) and final (chronic) day of exposures to predator cues and later analyzed for whole-body cortisol, glucose, lactate, and protein. Length and mass measurements were used to calculate condition factor of fish in each treatment. Turbidity had the greatest effect on juvenile delta smelt and resulted in reduced cortisol, increased glucose and lactate, and greater condition factor. Elevated temperatures reduced available energy in delta smelt, indicated by lower glucose and total protein, whereas predator cue exposure had negligible effects on their stress response. This is the first study to show reduced cortisol in juvenile delta smelt held in turbid conditions and adds to the growing data that suggest this species performs best in moderate temperatures and turbidities. Multistressor experiments are necessary to understand the capacity of delta smelt to respond to the multivariate and dynamic changes in their natural environment, and results from this study should be considered for management-based conservation efforts.

## Teaser

Juvenile delta smelt displayed decreased stress and increased energy and condition factor when exposed to higher turbidities (10–11 nephelometric turbidity units [NTU]) compared with clearer waters (1–2 NTU). Increased temperatures (21 vs 17°C) resulted in reduced energy in fish. Predator cues had negligible effects on delta smelt’s stress response.

## Introduction

Coastal estuaries are among the most degraded ecosystems worldwide with rapid human population growth and increased industrialization and urbanization depleting >90% of species (Lotze *et al.,* 2006). This loss of biodiversity has triggered increased dead zones, disease outbreaks, and introduction of invasive species, reducing ecosystem resilience and recovery potential (Worm *et al.,* 2006). Due to the importance of estuaries and the organisms that inhabit them, large-scale restoration and supplementation strategies are being used to promote conservation (Cloern and Jassby, 2012; Moyle *et al.,* 2018). An understanding of the impacts of environmental change and degradation and how they affect the physiology and fitness of native species is critical for these efforts to be successful.

The San Francisco Estuary (SFE) is one of the largest estuaries in the United States and is comprised of the San Francisco Bay, San Pablo Bay, Suisun Bay, and the Sacramento-San Joaquin Delta (hereafter, Delta) (Service, 2007). The SFE supports >750 species of plants and animals and is considered an international hotspot of biodiversity (Myers *et al.,* 2000; Healey *et al.,* 2016). Starting in the 19th century during the California Gold Rush, the SFE underwent associated habitat modifications, including extensive infrastructure to support increasing water demands (Nichols *et al.,* 1986). The Delta currently supplies water to >20 million California residents, and water diversions consume between 35 and 65% of the annual freshwater outflow, depending on the season (Sommer *et al.,* 2011). As a result of continued population growth and development, increased water diversions have caused significant damage to the SFE and the organisms within it.

The delta smelt (*Hypomesus transpacificus*) is a small, semi-anadromous fish endemic to the upper SFE (Moyle, 2002; Moyle *et al.,* 2018; Yanagitsuru *et al.,* 2022). Due to their exceptionally sensitive nature and unique life history, delta smelt have become an indicator species for ecosystem health (Moyle *et al.,* 2016, 2019). Most delta smelt live a year or less and have low fecundity (1200–2600 eggs per female) (Moyle *et al.,* 1992; LaCava *et al.,* 2015). Consequently, the species is vulnerable to even a single year of poor recruitment. Once a very abundant fish, delta smelt are now rare and the species is on the brink of extinction (Bennett, 2005; Hobbs *et al.,* 2017; Moyle *et al.,* 2018). Rapid declines in delta smelt population began in the 1980s and resulted in its listing as threatened under the State and Federal Endangered Species Acts in 1993 (U.S. Office of the Federal Register, 1993). The delta smelt population has continued to decrease, with all abundance indices pointing downwards since 2002, coinciding with declines in several other pelagic fishes in the upper estuary (Thomson *et al.,* 2010; Hobbs *et al.,* 2017). This phenomenon is known as the ‘Pelagic Organism Decline’ and much like delta smelt’s decline, is not a consequence of one single variable, but rather the cumulation of multiple anthropogenic factors altering the SFE (Sommer *et al.,* 2007; Brooks *et al.,* 2012).

Alterations to the estuary due to water diversions and climate change include, but are not limited to, habitat loss, reduced turbidity, higher salinity and temperature, and increased predation and competition caused by the introduction of invasive species (Moyle *et al.,* 2016, 2018; Hobbs *et al.,* 2017). These changes have negatively impacted delta smelt abundance, as these fish are commonly associated with the low salinity zone (<6 psu), moderate temperatures (7–25°C), and mid-turbid waters (10–50 NTU) (Swanson *et al.,* 2000; Feyrer *et al.,* 2007; Nobriga *et al.,* 2008; Sommer and Mejia, 2013). Understanding how habitat degradation and change affect the physiology and performance of delta smelt is imperative for setting environmental regulations and offering recommendations for rearing and supplementation of the wild population (Yanagitsuru *et al.,* 2022).

Turbidity is an understudied but critical variable affecting delta smelt life history, physiology, and survival. Turbidity is caused by the scattering and absorption of light by suspended particles and is often referred to as the ‘cloudiness’ of water (Kirk, 1985; Henley *et al.,* 2000). The interaction of light intensity, water depth, and the physical properties of suspended material all contribute to the intensity and effect of turbidity (Davies-Colley and Smith, 2001). Turbidity can have significant effects on ecosystems, such as alterations to species compositions and trophic interactions in estuaries (Lunt and Smee, 2014). It can also have both positive and negative effects in fish, depending on its intensity and the species of fish in question (Utne-Palm, 2002; Hasenbein *et al.,* 2016). Low levels of turbidity are often associated with reduced feeding and growth rates, increased predation, and higher levels of stress in fish (Boehlert and Morgan, 1985; Rieger and Summerfelt, 1997). High turbidity, on the other hand, may also lead to reduced feeding, clogged and damaged gills, diminished visual acuity, and increased entrainment (Gardner, 1981; Hecht and Van der Lingen, 1992; Sutherland and Meyer, 2007; Grimaldo *et al.,* 2009; Hess *et al.,* 2017). Turbidity in the SFE has declined significantly over the last couple of decades with a 36% decrease in suspended sediment concentration between years 1991–1998 and 1999–2007, due in part to sediment trapping in reservoirs and dams, riverbank protection, and depletion of erodible sediment from hydraulic mining (Wright and Schoellhamer, 2004; Schoellhamer, 2011). Decreased turbidity is thought to be one of several factors contributing to the Pelagic Organism Decline and the population collapse of delta smelt (Sommer *et al.,* 2007).

High temperatures and the increased occurrence of heat waves present a significant threat to the future of delta smelt (Moyle *et al.,* 2016). Water temperature projections coupled with thermal sensitivity metrics suggest that temperatures in the SFE will result in both lethal and sublethal effects in delta smelt, causing an overall reduction in its population and a substantial compression of their habitat (Brown *et al.,* 2016; Hung *et al.,* 2022b). In addition, critical life history events, such as the spawning and migration of delta smelt, are known to coincide with the most widespread temperature increases in the SFE (Bashevkin *et al.,* 2022). Habitat change due to climate change and water diversions has made the estuary more desirable to competitors and predators, such as the overbite clam (*Potamocorbula amurensis*), Asian clams (*Corbicula fluminea*), and warm-water fishes such as the largemouth bass (*Micropterus salmoides*) and Mississippi silversides (*Menidia beryllina*) (Kimmerer, 2006; Moyle *et al.,* 2016; Baumsteiger *et al.,* 2017; Komoroske *et al.,* 2020). These non-native and filter-feeding clams are responsible for significant water clearing, thus likely contributing to reductions in turbidity in the SFE (Carlton *et al.,* 1990; Paganini *et al.,* 2010). Introduction of invasive submersed aquatic vegetation (SAV), such as the Brazilian waterweed (*Egeria densa*), has slowed water movement, further increasing temperature and reducing turbidity (Durand, 2015). In addition, this SAV has reduced the pelagic habitat preferred by delta smelt and increased their risk of predation by introducing vegetated areas that predators such as the largemouth bass can use to ambush open-water prey (Ferrari *et al.,* 2014; Conrad *et al.,* 2016).

Based on climate change projections, industrial competition for water usage, and knowledge of their physiological vulnerabilities and complex life histories, delta smelt are likely to go extinct in the wild in the next 1–5 years without effective and immediate management intervention (Brown *et al.,* 2013, 2016; Komoroske *et al.,* 2014; Jeffries *et al.,* 2016; Moyle *et al.,* 2018, 2019; Hobbs *et al.,* 2019; Bork *et al.,* 2020). Consequently, current research aimed to conserve the delta smelt has shifted to focus on informing hatchery-based supplementation, which was initiated in winter 2021–2022 (Yanagitsuru *et al.,* 2022). The UC Davis Fish Conservation and Culture Laboratory (FCCL) is currently rearing a genetically managed refuge population for the purpose of both research and safeguarding the species against extinction (Baskerville-Bridges *et al.,* 2005; Fisch *et al.,* 2013; Lindberg *et al.,* 2013). As part of genetic management of the cultured delta smelt population, FCCL has been permitted to collect 100 wild adult delta smelt every year. In the last couple of years, this goal has become increasingly challenging to meet. Only two wild delta smelt were captured during the 2020–2021 season, exemplifying the need for rapid and efficient supplementation. The first experimental release of delta smelt occurred between December 2021 and February 2022 and the second-year release started in November 2022 (USFWS, 2019, 2020). To be successful in these efforts, physiological data from controlled laboratory experiments can help us gain a better understanding of delta smelt’s tolerances to key environmental parameters, such as temperature, turbidity, and predation by non-native species. This in turn can provide crucial information when selecting optimal transportation conditions and release environments to minimize stress and maximize survival in this highly sensitive species (Swanson *et al.,* 1996; Lessard *et al.,* 2018).

The objective of this study was to investigate the individual and combined effects of turbidity, temperature, and predator cues on the physiology and stress response of juvenile delta smelt. We hypothesized that delta smelt would display reduced stress and greater available energy in conditions with higher turbidity, lower temperature, and in the absence of predation cues. We also hypothesized that reduced temperatures and higher turbidities have mitigating effects on stress induced by predator cue exposure. To test these hypotheses, juvenile delta smelt were held at two temperatures (17 and 21°C) and two turbidities (1–2 and 10–11 , NTU) for a week before exposure to a largemouth bass predator cue. Delta smelt were then exposed to a similar largemouth bass predator cue at the same time every day for 7 days to compare the effects of an acute versus a chronic predation stress at different temperatures and turbidities. At the end of both the acute and chronic exposures, fish were measured and sampled for quantification of whole-body cortisol, glucose, lactate, protein, and condition factor. Maintaining homeostasis after stressor exposure is energetically expensive and can result in reduced aerobic scope and reduced energy available to allocate to basic physiological needs, such as metabolism, growth, and reproduction (Pörtner, 2001, 2010; Sokolova *et al.,* 2012). Therefore, the long-term effects of elevated stress on fish can be detrimental to their survival and result in population-level effects. A greater understanding of how environmentally relevant variables affect delta smelt’s stress response and physiology will help identify optimal conditions for rearing, transportation, and supplementation, and in turn, reduce risk of its extinction in the wild.

## Methods

### Study species and maintenance

Juvenile delta smelt (42.7 ± 0.3 mm fork length, 0.53 ± 0.01 g) were obtained from the University of California Davis FCCL in Byron, CA. Information about the design of the aquaculture facility, captive breeding program, and genetic management plan can be found in Lindberg *et al.* (2013), Tigan *et al.* (2020) and Hung *et al.,* (2021). In June 2021, delta smelt (~160 days post-hatch, dph) were transferred from FCCL to the UC Davis Putah Creek Aquaculture Facility (PCF). Fish were immediately introduced into 36, 15-gallon black polyethylene tubs (hereafter, sub tanks), each covered with circular lids made from shade cloth to avoid water contamination, prevent fish escapement, and to manage light intensity. The PCF includes a recirculating aquaculture system with external temperature control units and 12, 400-L tanks (hereafter, holding tanks) that function as experimental water baths. Each holding tank housed three sub tanks, with 30 fish per sub tank (Supplementary Fig. S1).

To optimize holding conditions and maintain turbidities, outdoor reservoir tanks with treatment water provided flow-through water to sub tanks. Excess water in sub tanks overflowed through a 1-inch hole covered in mesh to prevent fish from escaping. In addition, each sub tank contained its own external biofiltration unit, where water in the sub tanks was pushed through the unit filled with k1 biomedia and returned to the sub tanks using an airlift mechanism. The airlift unit also maintained oxygen levels by introducing freshly oxygenated water into sub tanks.

Delta smelt were acclimated to ambient conditions of 17°C and 1.4 NTU for 2 weeks before the start of experimentation (Supplementary Table S1). Fish were fed dry feed [BioVita starter (50% of #0 crumble and 50% #1 crumble)] at 2% of their body weight per day during two separate feedings and exposed to a natural photoperiod (12 L:12 D). Light was provided by indoor fluorescent light bulbs and light intensity was kept at a constant low level (55.74 lx ± 1.0) to match culture protocols (Lindberg *et al.,* 2013). Light intensity was measured using a portable digital light meter (LX1330B; Drmeter) to ensure values were consistent between sub tanks. To monitor water quality, daily temperature (°C), dissolved oxygen (mg/L), and salinity (psu) readings were taken using a handheld YSI 556 MPS meter (YSI Inc., Yellow Springs, OH) and turbidity using a Hach 2100q portable turbidity meter (Hach Company, Loveland, CO). pH was measured every other day using a pinpoint pH monitor (American Marine Inc., Ridgefield, CT). Ammonia, nitrate, and nitrite were measured two to three times a week using a Hach pocket colorimeter (Hach Company, Loveland, CO) for ammonia and a marine care multitest kit (Red Sea, Houston, TX) for ammonia, nitrite, and nitrate. Mortality was quantified daily, and any dead fish were immediately removed from sub tanks to maintain water quality. All handling, care, and experimental procedures used were reviewed and approved by the UC Davis Institutional Animal Care and Use Committee (IACUC Protocol 16 591).

**Table 1:** Condition factor data for juvenile delta smelt held at different temperatures (17 or 21°C) and turbidities (1–2 or 10–11 NTU) and exposed to an acute or chronic predator cue. Values are presented as mean ± SEM

**Temperature (°C)**	**Turbidity (NTU)**	**Timing**	**Condition Factor**
17	1–2	Acute	0.61 ± 0.01
21	1–2	Acute	0.58 ± 0.01
17	10–11	Acute	0.64 ± 0.01
21	10–11	Acute	0.61 ± 0.01
17	1–2	Chronic	0.63 ± 0.01
21	1–2	Chronic	0.64 ± 0.01
17	10–11	Chronic	0.66 ± 0.01
21	10–11	Chronic	0.65 ± 0.01

### Temperature and turbidity treatments

After the 2-week acclimation period (17°C and 1.4 NTU), delta smelt were exposed to temperatures of 17 or 21°C and turbidities of 1–2 or 10–11 NTU for a week before predator cue introduction. Temperatures and turbidities were gradually increased (1.3°C and 3 NTU per day) from acclimation levels to treatment levels over the course of 3 days. To reach desired turbidities, *Nannochloropsis* algae (Nanno 3600—High-yield grow-out feed; Reed Mariculture Inc., USA) were spiked into individual sub tanks. This algal suspension is also used by FCCL to increase turbidities when rearing larval delta smelt (Tigan *et al.,* 2020). To maintain treatment turbidity throughout the experimental period, *Nannochloropsis* algae were added to reservoir tanks connected to individual sub tanks via PFA standard tubing (inner diameter = 0.5 cm) connected to a standpipe located in the middle of each sub tank, allowing for daily introduction of fresh algae-spiked water. Salinity (1.6 ± 0.14 psu) was maintained in a similar manner by adding Instant Ocean (Aquarium Systems, Mentor, OH) to outdoor reservoir tanks so that fresh saline water was added via the same PFA standard tubing. Salinity levels were chosen to reflect conditions delta smelt are commonly associated with in their natural habitat (Bennett, 2005). In addition to daily temperature measurements, 12 HOBO temperature loggers were exchanged between sub tanks every couple of days and recorded temperatures (°C) every 15 min. All acclimation and treatment temperature and turbidity data are presented as mean ± standard error of the mean (SEM) in Supplementary Table S1.

### Predator cue

To test the effects of different temperatures and turbidities on both the acute and chronic predator stress response of juvenile delta smelt, a largemouth bass predator cue was introduced to sub tanks every day for 8 days. Predator cues were inserted at the same time each day (~1300), 3 h after the last feeding. Fish were then sampled 15 min after cue insertion on both the first (day 1) and last (day 8) day to assess the generalized stress response to acute and chronic predator cue exposure, respectively. Previous experiments on juvenile delta smelt confirmed that 15 min was sufficient timing for fish to display increased cortisol after a predator cue (Pasparakis *et al.,* 2022). Details on the preparation and insertion of predator cues are outlined in Pasparakis *et al.,* 2022. Briefly, predator cues were prepared the day before trials by housing a largemouth bass in an insulated and aerated cooler for 24 h with 50 mL of water per gram of bass. Cues were injected through PFA standard tubing (inner diameter = 0.5 cm) outside holding tanks with closed lids over sub tanks to avoid disturbance to fish. Tubing was fastened to sub tanks via zip ties so that cues were introduced 1 cm below water level and 3 cm downstream of outflow valves to maximize distribution. To prime the tubing, avoid air bubbles, and ensure all the predator cue was effectively pushed through tubing, 60 mL of tank water was injected before and after the 60 mL of predator cue. Control tanks received three 60-mL syringes of tank water. Fish were netted from individual sub tanks and immediately euthanized with an overdose of tricaine methanesulfonate (MS-222; Finquel) (500 mg/L MS-222; Finquel) buffered to a neutral pH with sodium bicarbonate. Fish were weighed, measured, and snap frozen in liquid nitrogen within <3 min after netting to ensure cortisol levels were not affected by handling stress. Fork length (mm) and wet mass (g) data for juvenile delta smelt measured on sampling days are presented in Supplementary Table S2.

## Biochemical Analysis of Stress Response

### Whole-body homogenization

A total of 144 delta smelt (4–5 replicates per treatment and 4 fish per replicate) from both the acute and chronic predator stress trials were analyzed for whole-body cortisol, glucose, lactate, and protein concentrations. Due to the complex dynamics of the hypothalamic-pituitary-interrenal axis and resulting variability in cortisol levels, the head of each frozen fish was cut off with a sterile razor blade. The remaining tissue was ground into a fine powder using a mortar and pestle over liquid nitrogen. Whole-body fish powder was weighed and then homogenized in 4 mL ice-cold 1× phosphate-buffered saline [PBS buffer: 137 mM sodium chloride, 2.7 mM potassium chloride, 10 mM disodium phosphate and 1.8 mM monopotassium phosphate (pH = 7.4)] and protease inhibitors (Roche Molecular Systems, Inc.), using a handheld homogenizer. The homogenate was then split into four equal parts for analysis of cortisol, glucose, lactate, and protein. Glucose, lactate, and protein samples were centrifuged for 30 min at 14 500*g* at 4°C, and supernatant was extracted and stored at −80°C for later analysis.

### Cortisol extraction and analysis

Cortisol extraction was performed following methodology outlined in Pasparakis *et al.,* 2022. Briefly, 1 mL of homogenate was transferred to a 9-mL Pyrex glass tube on the same day as tissue homogenization. Tissue homogenate was spiked with 2.5 mL of diethyl ether, vortexed for 1 min and then centrifuged for 7 min at 3200*g* at 4°C. Without touching the pellet, supernatant was extracted and transferred to a new 9-mL Pyrex glass tube. This process was repeated two more times for maximal cortisol extraction, and extracted supernatant from all three washes was combined. The pellet was then discarded, and the combined supernatant was left in a fume hood overnight to ensure full evaporation of diethyl ether. On the following day, samples were resuspended in 200 μL 1× PBS, vortexed and stored at −80°C until later analysis. Cortisol analysis was performed using an enzyme immunoassay (EIA) kit, following the manufacturer’s instructions (Salivary Cortisol Immunoassay, Salimetrics LLC). Samples were run in duplicate, and cortisol concentrations (μg·dL^−1^) were calculated using a four-parameter sigmoid standard curve. Cortisol levels were normalized to both mass (ng cortisol per g fish) and total protein (pg cortisol per μg protein) of each sample. Cortisol values corrected by fish mass were used in statistical analysis.

### Glucose and lactate analyses

Frozen tissue homogenate samples were thawed on ice, and analyses were conducted using commercial kits according to the manufacturer’s instructions for both glucose (glucose assay kit, Sigma-Aldrich) and lactate (lactate assay kit II, Sigma-Aldrich). Samples were run in duplicate, concentrations (ng·μL^−1^) were calculated using a linear standard curve and values were normalized to fish mass (μg·g^−1^).

### Protein to mass ratios

Protein concentrations were determined using the bicinchoninic acid method (Pierce, Thermo Fisher Scientific Inc.) following the manufacturer’s instructions. Samples were diluted 5-fold with 1× PBS buffer to match the kit’s serum albumin standards and then run in duplicate. Protein concentration (μg·mL^−1^) was calculated using a linear standard curve. Protein-to-mass ratios were calculated by dividing total protein (μg·mL^−1^) by mass of fish (g).

Samples outside the range of standard curve values for all four assays were eliminated from analyses. Standard curves of all assays had r^2^ values ≥0.99.

### Condition factor

Mass and fork length measurements of delta smelt were taken on both sampling days, during the acute and chronic predation stress exposures (Supplementary Table S2). These measurements were used to calculate Fulton condition factor using the equation CF = (W_B_/FL^3^) × 100, where W_B_ is the body mass (g) and FL is the fork length (cm).

### Statistical analysis

Statistical analyses were conducted using R version 4.1.2 (R Core Team, 2021), with the packages ‘nlme’ (Pinheiro *et al.,* 2019) and ggplot2 (Wickham, 2009). Non-parametric tests were used due to the necessity of including random effects, i.e. to account for effects of sub tanks. Linear mixed-effect models (LMEs) that incorporated sub tank as a random effect were used to analyse whole-body cortisol, glucose, and lactate data, protein-to-mass ratios, and condition factor. Fixed effects included temperature, turbidity, predator cue, and the timing of that cue (acute vs chronic). Multiple LMEs using singular, combined, and interactive effects of biological relevance were run, and Akaike information criterion (AICc) were calculated to determine the model of best fit for the data. Statistical output for these models can be found in Supplementary Table S3, and AICc scores are reported in Supplementary Table S4. The full LME was the most parsimonious model for glucose, lactate, and protein-to-mass ratios. The model exploring the interaction of turbidity and largemouth bass predator cue followed by the full model were the best fit models for cortisol data, whereas the model exploring timing of cue alone followed by the interaction of turbidity and timing were the most parsimonious models for condition factor (Supplementary Tables S3 & S4). Data were tested for normality and homogeneity using the ‘shapiro.test’ function from the stats package and the ‘leveneTest’ function from the ‘car’ package, respectively. The Kruskal-Wallis rank sum test was used to test the effect of water conditions (temperature and turbidity) on delta smelt mortality. Data are presented as means ± SEM, and differences between means were deemed significant at *P* < 0.05.

## Results

### Mortality

There was no effect of temperature nor turbidity on delta smelt survival (Kruskal-Wallis: χ^2^ = 5.93; *P* = 0.12), suggesting that these experimental treatments only induced sublethal effects to the fish tested.

### Cortisol

**Figure 1:**
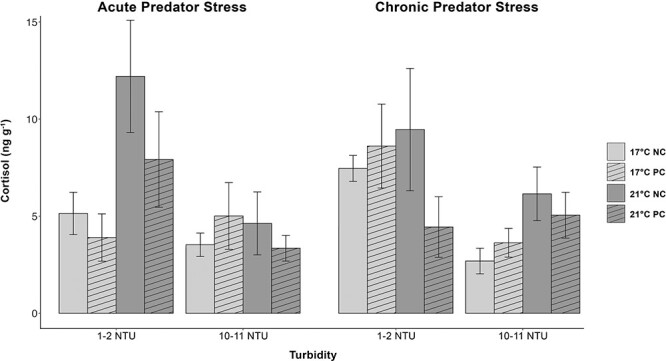
Whole-body cortisol measurements (ng·g^−1^) in juvenile delta smelt during an acute and chronic predator cue exposure (NC = no cue; PC = predator cue) held at two temperatures (light grey = 17°C; dark grey = 21°C) and two turbidities (1–2 NTU & 10–11 NTU). Fish were sampled 15 min after exposure to a largemouth bass predator cue (vertical angled bars) or tank water (blank bars). Cortisol levels were significantly lower in fish held in turbid compared with low-turbidity conditions. Data (*n* = 14–20) are presented as mean ± SEM.

There was a significant effect of turbidity on whole-body cortisol in juvenile delta smelt such that fish displayed reduced cortisol at higher turbidities (10–11 NTU) compared with lower turbidities (1–2 NTU) (*P* < 0.01) (Fig. 1). There was no effect of temperature, predator cue, or time point (acute vs chronic predator cue) on cortisol values. There were also no significant interactions of any fixed effects (Supplementary Table S3).

### Glucose

**Figure 2:**
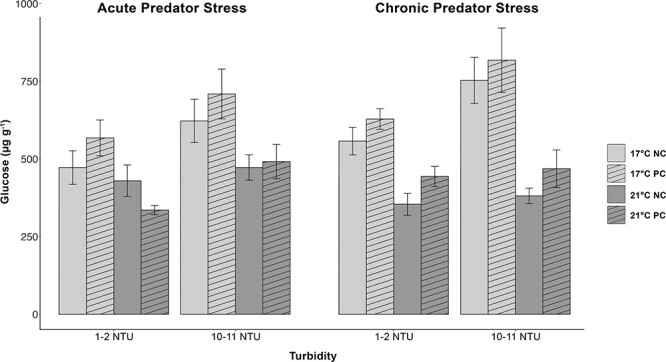
Whole-body glucose measurements (μg·g^−1^) in juvenile delta smelt during an acute and chronic predator cue exposure (NC = no cue; PC = predator cue) held at two temperatures (light grey = 17°C; dark grey = 21°C) and two turbidities (1–2 NTU & 10–11 NTU). Fish were sampled 15 min after exposure to a largemouth bass predator cue (vertical angled bars) or tank water (blank bars). Glucose levels were significantly greater in fish held at lower temperatures and greater turbidities. Data (*n* = 12–20) are presented as mean ± SEM.

Both temperature (*P* < 0.0001) and turbidity (*P* < 0.001) had a significant effect on whole-body glucose in delta smelt such that fish displayed increased glucose at 17°C compared with 21°C and at higher turbidities (10–11 NTU) compared with lower turbidities (1–2 NTU) (Fig. 2). There was no effect of predator cue, time point, or interaction of fixed effects on glucose levels (Supplementary Table S3).

### Lactate

**Figure 3:**
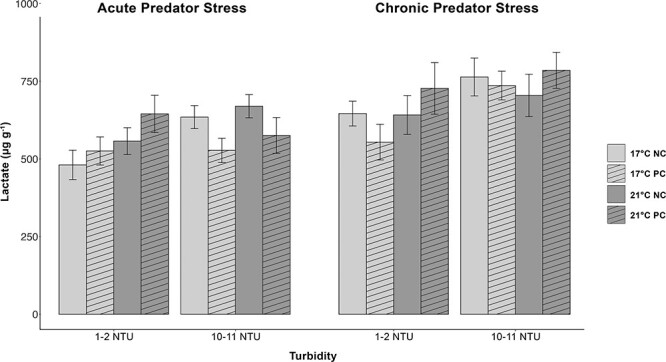
Whole-body lactate measurements (μg·g^−1^) in juvenile delta smelt during an acute and chronic predator cue exposure (NC = no cue; PC = predator cue) held at two temperatures (light grey = 17°C; dark grey = 21°C) and two turbidities (1–2 NTU & 10–11 NTU). Fish were sampled 15 min after exposure to a largemouth bass predator cue (vertical angled bars) or tank water (blank bars). Lactate levels were significantly greater in fish held in turbid conditions and during the chronic predator stress. Data (*n* = 14–20) are presented as mean ± SEM.

There was a significant effect of both turbidity (*P* < 0.005) and time point (*P* < 0.0001) on whole-body lactate in delta smelt such that fish displayed higher lactate levels when held at higher turbidities (10–11 vs 1–2 NTU) and during the chronic predator cue time point compared with the acute predator time point a week earlier (Fig. 3). There was no effect of temperature, predator cue, or interaction of fixed effects on lactate levels (Supplementary Table S3).

### Protein-to-mass ratio

**Figure 4:**
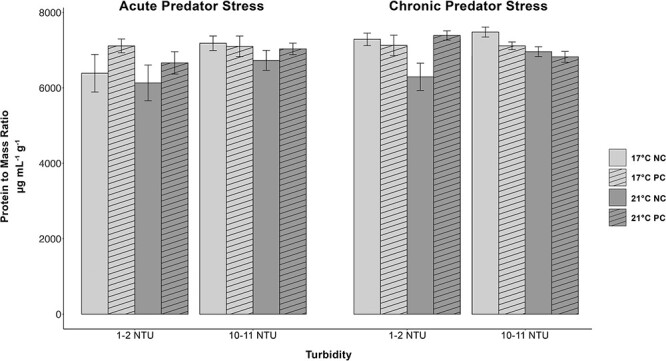
Protein-to-mass ratios (μg·mL^−1^·g^−1^) in juvenile delta smelt during an acute and chronic predator cue exposure (NC = no cue; PC = predator cue) held at two temperatures (light grey = 17°C; dark grey = 21°C) and two turbidities (1–2 NTU & 10–11 NTU). Fish were sampled 15 min after exposure to a largemouth bass predator cue (vertical angled bars) or tank water (blank bars). Protein-to-mass ratios were significantly greater in fish held at 17°C compared with those held at 21°C. Data (*n* = 15–20) are presented as mean ± SEM.

Delta smelt held at 17°C had significantly higher protein-to-mass ratios compared with fish held at 21°C (*P* < 0.05) (Fig. 4). There was no effect of turbidity, predator cue, or time point on protein-to-mass ratios. There were also no significant interactions of fixed effects (Supplementary Table S3).

### Condition factor

Condition factor in juvenile delta smelt was affected by both timing (acute vs chronic) (*P* < 0.0005) and turbidity (*P* < 0.05). Condition factor increased from acute to chronic exposures and decreased at lower turbidities (Table 1). There was no interaction of turbidity and timing (Supplementary Table S3).

## Discussion

The goal of this study was to explore how environmental changes, such as decreased turbidity, higher temperature, and greater predation affected the stress response and physiology of delta smelt. Juvenile delta smelt were exposed to varying temperatures (17 and 21°C) and turbidities (1–2 and 10–11 NTU) for 2 weeks before the introduction of an acute and chronic largemouth bass predator cue. Turbidity had the greatest effect on delta smelt, and fish held in more turbid conditions displayed reduced stress, greater condition factors, and increased energy in the form of both whole-body glucose and lactate. Lower temperature resulted in a significant increase in both whole-body glucose and protein-to-mass ratios. Finally, a largemouth bass predator cue had negligible effects on delta smelt.

Chronic stress and reduced energy can have significant impacts on populations of fish (Wendelaar Bonga, 1997). During stress, energy necessary for regaining homeostasis and inducing defense mechanisms is diverted away from growth and development (Pankhurst, 2011). Prolonged stress and increased cortisol have also been correlated to reduced immune function and increased susceptibility to disease (Tort, 2011). Reductions in growth and development and suppression of the immune system can ultimately lead to reduced fitness, fecundity, and survival in nature (Barton and Iwama, 1991; Barton, 2002). The results of this study add valuable information that can inform rearing conditions in aquaculture and release environments for supplementation of delta smelt.

### Turbidity

The importance of turbidity for the sustainability of delta smelt populations is becoming increasingly apparent. It is likely an important factor mediating the completion of their life cycle because increased pulse turbidity during the ‘first flush’ of the rainy season is thought to act as a cue for their annual spawning migration (Grimaldo *et al.,* 2009; Sommer *et al.,* 2011; Bennett and Burau, 2015). Similar to protocols at FCCL, our turbidity treatments were maintained using *Nannochloropsis* algae (Tigan *et al.,* 2020). Turbidity in the SFE is made up of a complex and variable mixture of suspended sediment, dissolved organic matter, and algae; therefore, our turbidity treatments are not representative of all combinations of environmental conditions. However, our results correspond well to field observations, where delta smelt are commonly found in mid-turbid waters ranging from 10 to 50 NTU. (Feyrer *et al.,* 2007). In our study, we chose to test two turbidities: a low-turbidity treatment of 1–2 NTU, reflecting reductions in turbidity due to environmental degradation and increased prevalence of invasive species, and a turbid treatment of 10–11 NTU, representative of delta smelt’s preferred habitat (Schoellhamer, 2011). These turbidities were chosen to facilitate comparisons with similar peer-reviewed studies, reflect ecological relevance, and to consider feasibility of maintenance in an aquaculture setting. The maintenance of turbidity in aquaculture is both time-consuming and expensive, and thus careful consideration must be made when assessing its cost–benefit analysis. Because there are limited data on the effects of turbidity on cultured juvenile delta smelt, a main goal of this study was to fill this knowledge gap.

Lower turbidity significantly increased cortisol levels, reduced metabolites glucose and lactate, and decreased condition factor in juvenile delta smelt over a 2-week exposure period (Fig. 1, Fig. 2, Fig. 3 and Table 1). These data suggest that juvenile delta smelt held in clearer waters of 1–2 NTU had increased stress, reduced available energy, and were in poorer condition than fish held at higher turbidities of 10–11 NTU. One possible explanation for these results is that turbidity influenced delta smelt’s feeding. Juvenile delta smelt are visual zooplankton feeders with a diet that consists of pelagic copepods, cladocerans, and mysid shrimp (Hobbs *et al.,* 2006; Moyle *et al.,* 2018). As visual feeders, their feeding success is determined by visual range and prey density (Hecht and Van der Lingen, 1992; Utne-Palm, 2002). In the current experiment, pellet density was kept consistent between sub tanks and visual range was affected by suspended particles or the turbidity in each treatment. Turbidity is thought to increase delta smelt’s visual acuity by enhancing the contrast between prey and its background and thus assist in feeding (Baskerville-Bridges *et al.,* 2004).

Although feeding rates were not quantified in this current study, previous studies show that delta smelt’s feeding success is greatly affected by turbidity (Baskerville-Bridges *et al.,* 2004; Hasenbein *et al.,* 2013, 2016; Tigan *et al.,* 2020). Larval delta smelt were found to have the highest feeding rates at 11 NTU, with a sharp decline in feeding at lower algal concentrations and lower light levels (Baskerville-Bridges *et al.,* 2004). Hasenbein *et al.* (2016) reported highest feeding rates in late-larval delta smelt between 25 and 80 NTU and reduced feeding at lower (5 and 12 NTU) and higher (120 and 250) turbidities, whereas juvenile delta smelt displayed reduced feeding at >250 NTU, consistent feeding between 12 and 120 NTU and highest feeding at <12 NTU (Hasenbein *et al.,* 2013). In the same studies (Hasenbein *et al.,* 2013, 2016), there were no documented effects of turbidity (0–250 NTU) on whole-body cortisol levels in juvenile delta smelt, but although non-significant, cortisol trends in late-larval delta smelt indicated minimal stress at mid-range turbidities (35–80 NTU) and elevated stress at low turbidities (5, 12 and 25 NTU). In addition, low and high turbidities resulted in reduced survival at the late-larval stage (Hasenbein *et al.,* 2016). Turbidity exposures in these experiments were performed over a 2-h period, likely explaining differences in our findings, which occurred over a 2-week period. Our study is the first, to our knowledge, to show that turbidity reduces stress in juvenile delta smelt (Fig. 1). These combined results suggest that reduced turbidity in the SFE may be having significant effects on delta smelt physiology and growth and likely contribute to population decline.

Whether a certain level of turbidity will provide benefit or harm to a fish is dependent on life stage, feeding style, intensity of turbidity, and other interacting environmental conditions. For example, turbidity resulted in reduced feeding rates of planktivorous bluegills (*Lepomis macrochirus*) and cape silversides (*Atherina breviceps)**,*** whereas the effect of turbidity on the spotted grunter (*Pomadasys commefsonnii*), a macrobenthivore, was not as pronounced (Gardner, 1981; Hecht and Van der Lingen, 1992). Turbidity (up to 160 NTU) had no effect on the feeding rate of juvenile rainbow trout (*Oncorhynchus mykiss*), which the authors suggest was accomplished by alternating senses of prey detection depending on turbidity conditions (Rowe *et al.,* 2003). Adult European smelt (*Osmerus eperlanus*) had the highest feeding rates at 20 NTU turbidity, irrespective of light levels (Horppila *et al.,* 2004). Turbidity enhanced the feeding and/or growth rates of larval Pacific herring (*Clupea pallasii*), Atlantic halibut (*Hippoglossus hippoglossus*), walleye (*Stizostedion vitreum*), and rainbow smelt (*O. mordax*) (Boehlert and Morgan, 1985; Naas and Harboe, 1992; Rieger and Summerfelt, 1997; Sirois and Dodson, 2000). Because there was no evidence of increased prey ingestion in rainbow smelt larvae held in turbid conditions, the authors attributed their greater growth to a reduction in energy expenditure (Sirois and Dodson, 2000).

In the current study, turbidities previously demonstrated as favorable (Feyrer *et al.,* 2007) maintained delta smelt in better condition, and low turbidities decreased energy stores in the form of free glucose and lactate (Table 1, Fig. 2 and Fig. 3). Lactate is a substrate of glucose and is known to increase its concentrations, likely explaining the correlation of these two metabolites (Suarez and Mommsen, 1987; Polakof *et al.,* 2012). Previous studies have attributed high glucose and lactate and lower cortisol to fish in better condition (Chase *et al.,* 2016; Pasparakis *et al.,* 2022). In support of this, delta smelt in the current study displayed lower cortisol, greater glucose and lactate, and increased condition factor in higher turbidities (Table 1). Turbidity may have reduced the energetic cost of locating food. Alternatively, decreased glucose and lactate may be linked to prolonged cortisol induction in fish held in clearer waters. Stress adaptation involves the relocation of metabolic energy toward the maintenance of homeostasis and away from processes such as growth and reproduction (Wendelaar Bonga, 1997). Fish under chronic stress frequently display reduced growth, and parameters such as condition factor and food conversion efficiency are common indicators of stress in fish (Barton and Iwama, 1991; Barton, 2002). Future studies investigating the effects of long-term turbidity exposures on the feeding and growth rates of juvenile delta smelt are needed.

Smelt are known to have light sensitivities and actively avoid light in their natural environments (Appenzeller and Leggett, 1995; Horppila *et al*., 2004). Preference for low-light conditions is found to ultimately influence smelt distribution (Dembiński, 1971; Heist and Swenson, 1983). Delta smelt in the current study did not have the option of evading greater light intensities in their sub tanks, providing another possible explanation for elevated cortisol at low turbidities. To reduce stress to fish at the FCCL, early-stage delta smelt larvae are reared at a turbidity of 5.5 NTU. Because delta smelt are believed to have life stage-dependent sensitivities to light, late-larval, juveniles, and adults are reared in clear waters. Reduced lighting provided by shade cloth was found to increase juvenile survival during their 3-day transition to outdoor tanks (Lindberg *et al.,* 2013). Results from the current study suggest that the maintenance of higher turbidity may provide benefit to juvenile delta smelt as well as larval and late-larval stages and thus should be considered for rearing. In addition, environments with moderate turbidities should be prioritized during delta smelt supplementation to reduce stress, preserve energy, and maximize survival.

### Temperature

Temperature is a critical determinant of a species’ fitness, performance, and distribution, and warming waters due to climate change have resulted in the range shifts and niche compressions of many aquatic organisms (Pörtner, 2002; Schulte *et al.,* 2011; Schulte, 2015). Laboratory experiments are a tool we can use to investigate the sublethal effects of warming temperatures on the physiological performance of a species of interest to understand how future climate projections will affect their survival and sustainability (Helmuth, 2009). In the current experiment, juvenile delta smelt held at an increased temperature of 21°C displayed significantly reduced energy in the form of both available glucose and protein compared with fish held at 17°C (Fig. 2 and Fig. 4). Optimal physiological performance typically occurs over a narrow range of temperatures, and temperatures above or below this range may impose a significant energetic cost on a species (Pörtner, 2002, 2010; Schulte *et al.,* 2011). Reductions in glucose and protein are likely a consequence of increased energy demand associated with maintenance of homeostasis and induction of thermal stress defense mechanisms in juvenile delta smelt.

Delta smelt are especially sensitive to high temperatures and have lower thermal tolerances than many other native and non-native fish in the SFE including green sturgeon (*Acipenser medirostris*), Sacramento splittail (*Pogonichthys macrolepidotus*), Mississippi silversides (*M. beryllina*), largemouth bass (*M. salmoides*), and some populations of Chinook salmon (*Oncorhynchus tshawytscha*) (Young and Cech Jr, 1996; Sardella *et al.,* 2008; Davis *et al.,* 2019a; Zillig *et al.,* 2020). Depending on life stage and acclimation temperature, the critical thermal maximum (CTMax) temperature in delta smelt has been found to range from 24°C to just under 30°C (Swanson *et al.,* 2000; Komoroske *et al.,* 2014; Jeffries *et al.,* 2016; Davis *et al.,* 2019a). Most juvenile and pre-adult delta smelt are caught at temperatures <24°C, and a mean daily temperature of 25°C has been identified as a threshold for high mortality (Swanson *et al.,* 2000; Nobriga *et al.,* 2008; Cloern *et al.,* 2011). Although these temperatures are important to consider for management regulations, sublethal critical thresholds have been found to occur much below (4–6°C) upper tolerance limits and likely have significant impacts on delta smelt’s population and future survival (Komoroske *et al.,* 2015; Jeffries *et al.,* 2016; Davis *et al.,* 2019b).

In line with results from the current study, juvenile delta smelt acutely exposed to 20°C displayed increased metabolic rate as well as increased expression of genes relating to metabolic processes, ion regulation, and protein synthesis, indicating that this temperature imposed a substantial energetic demand (Jefferies *et al*., 2016). Gene expression data suggests that delta smelt experience physiological stress 4–6°C below their upper tolerance limits at all life stages (Komoroske *et al.,* 2015). In addition, delta smelt displayed limited thermal acclimation capacity, suggesting they are unable to modify cellular mechanisms and physiology to cope with higher temperatures (Komoroske *et al.,* 2014, 2015). Upregulation of genes and protein products is energetically expensive, and continued upregulation despite sufficient acclimation periods implies that delta smelt are living close to their thermal maximum (Feder and Hofmann, 1999; Komoroske *et al.,* 2015). After a 7-day exposure at 21°C, juvenile delta smelt displayed elevated swimming velocities, further supporting the notion that temperatures several degrees below thermal thresholds incur an energetic cost and provide a possible explanation for reduced energy in delta smelt observed in the current study (Davis *et al.,* 2019b). These combined results suggest that to sustain delta smelt populations in the wild, management thresholds need to incorporate a thermal buffer several degrees below upper tolerance limits. In addition, supplementation release efforts should continue to occur in winter months when temperature stress is reduced.

### Predation

Turbidity is thought to act as a cover for and decrease predation of open water prey species such as delta smelt. Laboratory studies showed that largemouth bass predation on delta smelt was reduced in turbid conditions (Ferrari *et al.,* 2014). Field studies also found that turbidity was an important predictor of predation because delta smelt DNA was more frequently found in the stomachs of Mississippi silversides caught in non-turbid waters (Schreier *et al.,* 2016). Therefore, we predicted that a largemouth bass predator cue would induce a stress response in juvenile delta smelt, which would be mitigated by turbid conditions. Predator cue exposures had no effect on delta smelt’s primary or secondary stress response as indicated by negligible differences in whole-body cortisol, glucose, or lactate levels (Fig. 1, 2 and 3). However, we did find that there was a significant increase in whole-body lactate and condition factor after 7 days of chronic predator cue exposure compared with fish that were only acutely exposed for 1 day (Fig. 3, Table 1). Due to the lack of direct effects from acute predator cues, it is possible that increases in lactate and condition factor were a result of a week of extra growth in captivity versus a week of chronic predator cues.

Many species of fish respond to predation cues, whether visual, acoustic, or olfactory in nature, by inducing their primary stress response and increasing cortisol levels (Remage-Healey *et al.,* 2006; Barcellos *et al.,* 2007; Barreto *et al.,* 2014; Barkhymer *et al.,* 2019). However, in some cases, fish are found to display signs of increased stress in the absence of significant increases in cortisol (Hasenbein *et al.,* 2016; Miyai *et al.,* 2016). Late-larval delta smelt exposed to high turbidities of 250 NTU exhibited elevated mortality and gene expression suggestive of oxidative and osmotic stress but displayed surprisingly low levels of cortisol (Hasenbein *et al.,* 2016). Nile tilapia (*Oreochromis niloticus*) exposed to a predator odor displayed decreased activity and increased ventilation rate, indicative of increased stress, with no corresponding changes in plasma levels of cortisol or glucose (Miyai *et al.,* 2016). Although previous studies found a similar largemouth bass predator cue elicited a stress response in juvenile delta smelt, cortisol results from the current study suggest that this cue was not strong enough to increase stress in delta smelt (Pasparakis *et al.,* 2022). Alternatively, due to delta smelt’s limited tolerance to stress and known variability of cortisol results, it is possible that increased stress was not detected through whole-body cortisol or metabolite results in this study (Hasenbein *et al.,* 2013; Moyle *et al.,* 2016). Plasma cortisol levels may have revealed increased stress but this analysis was not possible due to the size of juvenile delta smelt. Future studies should investigate how predator and competitor cues from other species of fish affect delta smelt’s stress response.

The significant increase in lactate without corresponding increases in cortisol or glucose between acute and chronic predator stress exposures was an unexpected finding (Fig. 3). Increased lactate may indicate that chronic exposure to a predator cue affected delta smelt’s swimming behaviors, such that increased swim velocity resulted in a shift to anaerobic respiration and thus higher lactate production (Pankhurst, 2011). In support of this, juvenile delta smelt exposed to a largemouth bass predator cue displayed increased swimming speeds and other swim behaviors suggestive of increased stress (Davis *et al.,* 2019b).

An alternative explanation for the lack of results is that delta smelt used in this study were raised in captivity and did not recognize the largemouth bass odor as a predator cue. Because largemouth bass are an invasive species, it is possible that delta smelt born and raised in the SFE would perceive this odor as a threat due to learned associations, whereas delta smelt in the current study, without a reference, perceived this cue as innocuous. Because supplementation is now necessitated for the maintenance of the delta smelt population in the SFE, fish raised in captivity will become the new wild delta smelt. Therefore, understanding how hatchery-raised fish respond to environmental signals, such as a predation cue, is important and environmentally relevant for this endangered species.

## Concluding Remarks

The SFE is a complex ecosystem with many interacting variables that have contributed to the long-term population declines of delta smelt. Laboratory experiments incorporating a multistressor design can be used to better understand how individual stressors and their interactions will affect the physiology of a species in question. Temperature and turbidity were both important variables affecting the physiological stress response of juvenile delta smelt. Increased temperatures caused a reduction in delta smelt’s energy in the form of available glucose and protein, whereas lower turbidities resulted in reduced available glucose and lactate, increased cortisol, and decreased condition factor. As the frequency and severity of events such as heat waves and droughts increase due to climate change, the challenges associated with delta smelt conservation will continue to intensify. Increased water demands and habitat alterations, such as continued declines in turbidity and increases in temperature in the SFE, will further reduce the delta smelt population and compress their limited distribution (Nobriga *et al.,* 2008; Moyle *et al.,* 2016). Findings from the current study should be used to inform regulations and initiatives to prevent delta smelt extinction. Specifically, mid-range turbidities and temperatures should be prioritized in the rearing, transportation, and release of delta smelt. This will facilitate the supplementation of delta smelt with greater condition factor, increased energy, and reduced stress, favoring their chances of survival in nature.

## Funding

This research was made possible by funding from the U.S. Fish and Wildlife Service no. F19AC00943 to A.E.T., R.E.C., T-C.H. and N.A.F., and the University of California, Davis Agricultural Experiment Station (CA-D-ASC-2252-H to A.E.T. and CA-D-ASC-2098-H to N.A.F.). USBOR, R20AC00027 funding to T-C.H. was used for delta smelt rearing and maintenance. Funding for F.B. was provided by the Bayerische Forschungsstiftung Scholarship (DOK-181-19, Geist). The findings and conclusions in this article are those of the authors and do not necessarily represent the view of the U.S. Fish and Wildlife Service.

## Supplemental Data Section

Supplementary material is available at *Conservation Physiology* online. Data in this section include an experimental schematic figure, a table with temperature and turbidity (mean ± SEM) in different treatments and during the acclimation period, a table including length and mass data for juvenile delta smelt on sampling days, a table with the statistical output for linear mixed-effects models and a table with the AICc scores for determination of best fit LMEs.

## Author Contributions

This manuscript was written through contributions of all authors. All authors have given approval to the final version of the manuscript. C.P., D.E.C., R.E.C., N.A.F. and A.E.T. designed the experiment. C.P., T.L. and F.B. collected the data. E.W.C. contributed biological expertise and expertise in the management of delta smelt. T-C.H. provided experimental fish. C.P. wrote the manuscript. R.E.C., N.A.F. and A.E.T. secured the funding that supported the work.

## Data Availability

The data underlying this article are available in the online supplementary material.

## Supplementary Material

Web_Material_coad036
