# Supplementary material for: Effects of turbidity, temperature and predation cue on the stress response of juvenile delta smelt
Source: Conserv Physiol. 2023 Jun 26;11(1):coad036. doi: 10.1093/conphys/coad036 (PMC10295165; doi:10.1093/conphys/coad036)
Supplement: Web_Material_coad036 [file web_material_coad036.pdf]

Supplemental Data

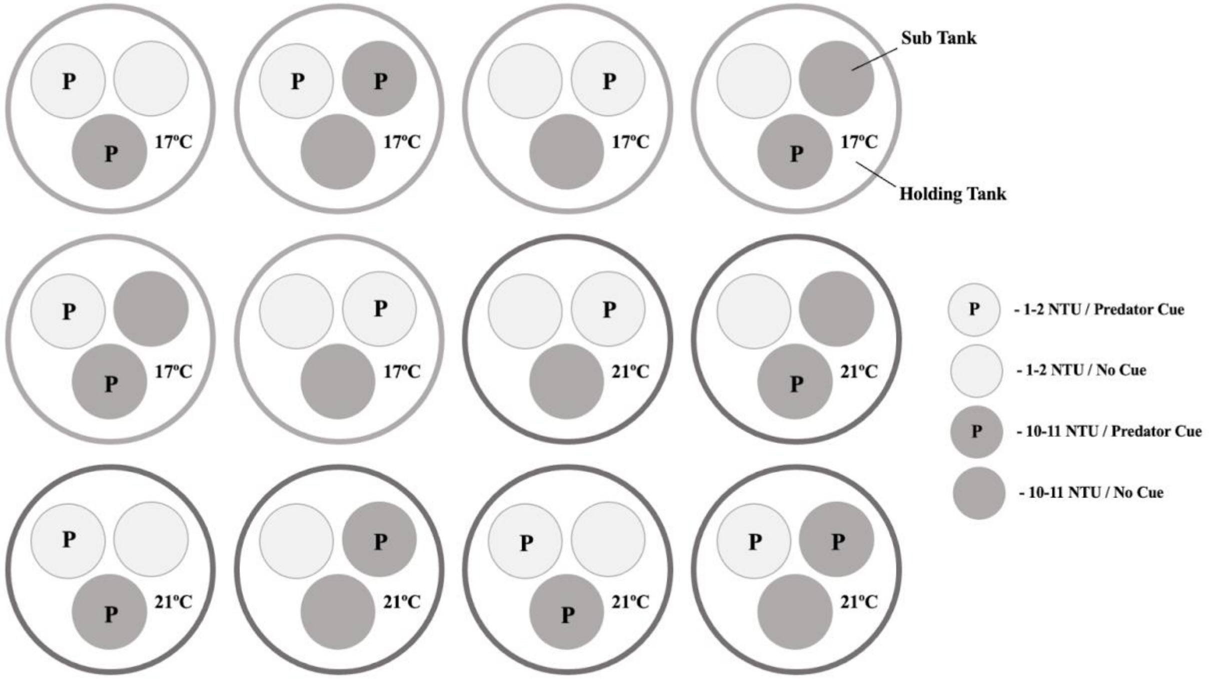

Supplemental Figure 1- Experimental schematic for effects of turbidity, temperature, and predation cues on the stress response in juvenile delta smelt. Three 15-gal sub tanks were placed in each 400 L holding tank for temperature control. Following a one-week acclimation to temperatures of 17 or 21°C and turbidities of 1-2 or 10-11 NTU, a largemouth bass (*Micropterus salmoides*) predator cue was introduced to sub tanks. Predator cues were inserted into sub tanks every day for eight days at the same time each day (~1300). To investigate the effects of an acute versus chronic predator stress in different environmental conditions, fish were sampled 15 minutes following cue insertion on the first and last day. Samples were analyzed for whole-body cortisol, glucose, lactate, and protein. Sub tanks were randomized within holding tanks. Image not to scale.

	Acclimation		Treatment	
**Treatment**	**Temp (ºC)**	**Turbidity (NTU)**	**Temp (ºC)**	**Turbidity (NTU)**
**17ºC; 1-2 NTU; NC**	16.98 ± 0.15	1.31 ± 0.08	16.79 ± 0.18	1.35 ± 0.09
**17ºC; 1-2 NTU; PC**	16.79 ± 0.10	1.09 ± 0.08	16.80 ± 0.11	1.15 ± 0.04
**21ºC; 1-2 NTU; NC**	17.00 ± 0.13	1.31 ± 0.07	20.43 ± 0.09	1.57 ± 0.20
**21ºC; 1-2 NTU; PC**	17.02 ± 0.18	1.25 ± 0.11	20.64 ± 0.07	1.09 ± 0.04
**17ºC; 10-11 NTU; NC**	16.91 ± 0.21	1.42 ± 0.10	16.94 ± 0.06	10.95 ± 0.17
**17ºC; 10-11 NTU; PC**	17.2 ± 0.18	1.43 ± 0.09	16.86 ± 0.20	11.20 ± 0.15
**21ºC; 10-11 NTU; NC**	16.90 ± 0.18	1.58 ± 0.03	20.44 ± 0.10	10.52 ± 0.21
**21ºC; 10-11 NTU; PC**	16.82 ± 0.09	1.37 ± 0.06	20.46 ± 0.03	11.02 ± 0.05

Supplemental Table 1. Temperature (ºC) and turbidity (NTU) during acclimation and treatment in all eight treatments distinguished by temperature, turbidity, and receival of predator cue (PC= predator cue; NC= no cue). Values are mean ± SEM.

Trial	Fork Length (mm)	Wet Mass (g)
Acute Predator Stress	41.80 ± 0.36	0.49 ± 0.01
Chronic Predator Stress	43.47 ± 0.35	0.57 ± 0.01

Supplemental Table 2. Fork length (mm) and wet mass (g) of juvenile delta smelt on sampling days for acute and chronic predator stress.

Cortisol (interaction)				
Fixed Effects	*Std.Error*	*df*	*t-value*	*p-value*
Turbidity	1.71	33	-2.78	0.009**
Predator cue	1.74	33	-1.5	0.1427
Interactive Effects				
Turbidity : Predator cue	2.44	33	1.12	0.2693
Cortisol (full)				
Fixed Effects	*Std.Error*	*df*	*t-value*	*p-value*
Temperature	0.3	33	1.43	0.1635
Turbidity	1.2	33	-2.81	0.0083**
Predator cue	1.2	33	-1.03	0.3098
Timing (acute vs chronic)	0.83	234	0.46	0.6453
Glucose (full)				
Fixed Effects	*Std.Error*	*df*	*t-value*	*p-value*
Temperature	7.47	33	-7.51	0.0000***
Turbidity	29.93	33	3.84	0.0005***
Predator cue	29.88	33	1.67	0.1043
Timing (acute vs chronic)	29.89	227	1.27	0.2046
Lactate (full)				
Fixed Effects	*Std.Error*	*df*	*t-value*	*p-value*
Temperature	6.86	33	1.85	0.0734
Turbidity	27.46	33	3.12	0.0038***
Predator cue	27.44	33	-0.26	0.7968
Timing (acute vs chronic)	27.44	241	4.29	0.0000***
Protein to mass (full)				
Fixed Effects	*Std.Error*	*df*	*t-value*	*p-value*
Temperature	40.66	33	-2.41	0.0217*
Turbidity	162.68	33	1.73	0.0925
Predator cue	162.64	33	1.45	0.1565
Timing (acute vs chronic)	126.12	248	1.88	0.0613
Condition factor (timing only)				
Fixed Effects	*Std.Error*	*df*	*t-value*	*p-value*
Timing (acute vs chronic)	0.007	843	5.03	0.0000***
Condition factor (interaction)				
Fixed Effects	*Std.Error*	*df*	*t-value*	*p-value*
Turbidity	0.03	34	2.28	0.0292*
Timing (acute vs chronic)	0.04	842	4.06	0.0001***
Interactive Effects				
Turbidity : Timing	-0.01	842	-0.7	0.4855

Supplemental Table 3. Statistical output for linear mixed effects models for whole-body cortisol, glucose, and lactate as well as protein to mass, and condition factor measurements. Sub tanks were included as random effects and fixed effects include temperature, turbidity, predator cue, and the timing of that cue (acute vs. chronic). The full model was the most parsimonious model for glucose, lactate, and protein to mass ratio data. The interactive model of turbidity and predator cue data followed by the full model were the most parsimonious for cortisol data, while the model exploring timing alone followed by the interaction of turbidity and timing were the best fit models for condition factor data. * = p<0.05; ** = p<0.01; *** = p<0.005.

Model	*df*	*AICc*
Cortisol		
Full	*7*	*1845.636*
Turbidity x Temperature	*6*	*1847.446*
Turbidity x Predator Cue	*6*	*1841.789*
Turbidity x Timing	*6*	*1845.36*
Temperature x Predator Cue	*6*	*1851.362*
Temperature x Timing	*6*	*1854.806*
Predator Cue x Timing	*6*	*1851.249*
Tubidity	*4*	*1845.73*
Temperature	*4*	*1853.776*
Predator Cue	*4*	*1851.964*
Timing	*4*	*1853.421*
Glucose		
Full	*7*	*3633.643*
Turbidity x Temperature	*6*	*3643.419*
Turbidity x Predator Cue	*6*	*3672.5*
Turbidity x Timing	*6*	*3674.021*
Temperature x Predator Cue	*6*	*3674.021*
Temperature x Timing	*6*	*3674.021*
Predator Cue x Timing	*6*	*3678.504*
Tubidity	*4*	*3689.686*
Temperature	*4*	*3670.536*
Predator Cue	*4*	*3694.789*
Timing	*4*	*3695.399*
Lactate		
Full	*7*	*3795.238*
Turbidity x Temperature	*6*	*3819.027*
Turbidity x Predator Cue	*6*	*3817.554*
Turbidity x Timing	*6*	*3799.743*
Temperature x Predator Cue	*6*	*3826.536*
Temperature x Timing	*6*	*3812.457*
Predator Cue x Timing	*6*	*3809.94*

Tubidity	*4*	*3832.69*
Temperature	*4*	*3841.394*
Predator Cue	*4*	*3841.623*
Timing	*4*	*3824.599*
Protein to Mass		
Full	*7*	*4770.067*
Turbidity x Temperature	*6*	*4786.318*
Turbidity x Predator Cue	*6*	*4780.09*
Turbidity x Timing	*6*	*4781.968*
Temperature x Predator Cue	*6*	*4785.036*
Temperature x Timing	*6*	*4786.714*
Predator Cue x Timing	*6*	*4783.523*
Tubidity	*4*	*4807.529*
Temperature	*4*	*4807.708*
Predator Cue	*4*	*4808.327*
Timing	*4*	*4807.225*
Condition Factor		
Full	*7*	*-1404.214*
Turbidity x Temperature	*6*	*-1385.569*
Turbidity x Predator Cue	*6*	*-1396.154*
Turbidity x Timing	*6*	*-1413.471*
Temperature x Predator Cue	*6*	*-1384.901*
Temperature x Timing	*6*	*-1406.741*
Predator Cue x Timing	*6*	*-1410.912*
Tubidity	*4*	*-1406.743*
Temperature	*4*	*-1400.432*
Predator Cue	*4*	*-1404.176*
Timing	*4*	*-1426.002*

Supplemental Table 4. Akaike information criterion (AICc) scores for singular, combined, and interactive fixed effects for determination of best fit linear mixed effect models for cortisol, glucose, lactate, protein to mass ratio, and condition factor data.
